# Anatomy and Biomechanics of Peltate *Begonia* Leaves—Comparative Case Studies

**DOI:** 10.3390/plants11233297

**Published:** 2022-11-29

**Authors:** Annabell Rjosk, Christoph Neinhuis, Thea Lautenschläger

**Affiliations:** Institute of Botany, Department of Biology, Technische Universität Dresden, 01062 Dresden, Germany

**Keywords:** anatomy, biomechanics, peltate leaves, *Begonia*, petiole-lamina transition zone

## Abstract

Plants are exposed to various external stresses influencing physiology, anatomy, and morphology. Shape, geometry, and size of shoots and leaves are particularly affected. Among the latter, peltate leaves are not very common and so far, only few studies focused on their properties. In this case study, four *Begonia* species with different leaf shapes and petiole attachment points were analyzed regarding their leaf morphology, anatomy, and biomechanical properties. One to two plants per species were examined. In all four species, the petiole showed differently sized vascular bundles arranged in a peripheral ring and subepidermal collenchyma. These anatomical characteristics, low leaf dry mass, and low amount of lignified tissue in the petiole point toward turgor pressure as crucial for leaf stability. The petiole-lamina transition zone shows a different organization in leaves with a more central (peltate) and lateral petiole insertion. While in non-peltate leaves simple fiber branching is present, peltate leaves show a more complex reticulate fiber arrangement. Tensile and bending tests revealed similar structural Young’s moduli in all species for intercostal areas and venation, but differences in the petiole. The analysis of the leaves highlights the properties of petiole and the petiole-lamina transition zone that are needed to resist external stresses.

## 1. Introduction

In general, the plant leaf is an important organ for photosynthesis and organic compound production [1,2]. To secure their function and orient themselves toward the light, leaves must be able to carry their own weight and twist and bend under external forces without being damaged [3]. Many of the diverse leaf shapes and forms can be mechanically described as a cantilevered beam (the petiole), fixed at the shoot supporting the lamina on the other side [3]. The stability and flexibility of the leaf are defined by the combination and spatial arrangement of tissue types within the petiole and lamina and their different mechanical properties [3,4]. The mechanical behavior of hydrostatic tissues, like the viscoelastic parenchyma and collenchyma, depends on turgor pressure [4]. The thin cell walls of parenchymatic tissue alone are mechanically of no great relevance, but in combination with turgor pressure internal and external loads are transferred as tensile stresses to the cell walls resulting in stiffening the tissue as a whole [4]. Not only turgor pressure affects the mechanical properties of parenchyma tissue, but also density of cell packaging [4]. Collenchyma tissue is found in growing organs, stems, and leaves and characterized by partially thickened cell walls and elongated cell shape [5]. Its elastic modulus is higher compared to parenchyma and additionally, its mechanical properties are affected by tissue age [4,6,7]. In contrast to hydrostatic tissues, sclerenchymatous tissue, such as fibers, xylem elements, and sclereids, have lignified, thickened cell walls [8]. The polymer lignin reinforces cell walls resulting in a higher elastic moduli and thus, is essential for the stiffness of organs [4,9].

While anatomy and biomechanics of leaves are generally well-studied so far, only few studies focused on peltate-shaped leaves and their properties. Peltate leaves are characterized by the petiole attaching to the lamina on the abaxial side of the leaf [10]. The petiole insertion point can be in the center of the leaf or closer to the leaf margin [10,11]. The petioles of peltate leaves are characterized by their unifacial anatomy [10,12,13]. Wilhelm Troll was one of the first to provide an explanation and definition of the peltate leaf shape and studied the morphology and anatomy of these leaves and their petioles [10]. In the 1950s, first studies on the development of peltate leaves and unifacial petioles were conducted [10,12]. In 1998, Friedrich Ebel followed with a detailed analysis regarding the geographical and habitat-specific distribution of peltate-leaved species [14]. After early attempts, a study among vascular plants, comprising approximately 350 peltate-leaved taxa representing 40 families and 99 genera was compiled [11]. Compared to the estimated number of vascular plants exceeding 380,000 species, peltate leaves are not very common [11,15]. Nonetheless, appearance and dimensions of peltate leaves are very diverse [10,11,14].

*Begonia* L. is one of the two genera of Begoniaceae and one of the largest and diverse genera of flowering plants, exceeding 1800 species in 70 sections [16,17]. The number of published species of the pantropical genus has increased considerably in the last decades and is estimated well over 2000 [16,17]. Begonias are terrestrial or epiphytic, perennial (rarely annual) herbs or shrubs [16] having herbaceous (often succulent) or woody stems or are tuberous and acaulescent. The leaves are arranged spirally and petiolate, typically asymmetric and diverse in shape and geometry [16]. In 21 of 70 sections peltate leaves can be found [16,17]. While there are some early general anatomical studies on *Begonia* [18,19], no detailed analysis has been conducted on the anatomy and biomechanical properties of peltate *Begonia* leaves in comparison to non-peltate *Begonia* species.

Contrary to the 2-dimensional architecture of leaves with a marginally attached petiole, peltate leaves are defined by a 3-dimensional spatial arrangement [20]. In particular the transition zone from petiole to peltate lamina shows a significant change in geometry [11,20]. To secure the connection between petiole and lamina, the transition zone needs to provide mechanical stability while transporting water and nutrients through petiole and lamina. The important role of the transition zone of peltate leaves in handling mechanical stress and load dissipation has already been demonstrated [21] and showed significant differences in mechanical properties of petiole and petiole-lamina transition zone indicating that petiole and transition zone are optimized to cope with different mechanical loads [22]. Additionally, in several studies different types of fiber organization in the transition zone were revealed [20,21]. A following anatomical study focusing on the fiber orientation in the transition zone from petiole to lamina in a variety of peltate plant species identified seven types of strengthening structures [11]. The transition zones of three *Begonia* species analyzed in this study were described as net-like structures.

In this case study, we aim to provide an analysis of morphology, anatomy, and biomechanics of the leaves of four *Begonia* species focusing on the petiole and the petiole-lamina transition zone to understand the principles behind tensile stiffness, tensile fracture mechanics and damage prevention of the leaf. The overall motivation for our studies of the transition zones of peltate leaves is the transfer of the obtained results into carbon-fiber reinforced concrete components. In a leaf, the fibers run through the petiole and into the lamina with the transition zone securely connecting these two leaf parts. This connection is load adapted and differently organized in different species. The peltate leaf can be seen as a model for future biomimetic junctions between a concrete column and a plate, such as a ceiling of a room. For a secure connection between concrete column and plate, a load-adapted structure is needed where the carbon-fibers are arranged in a way that helps load dissipation from the plate into the column. The differently organized petiole-lamina transition zones can be described anatomically and biomechanically and transferred into models that serve as inspiration for the carbon-fiber arrangement in column-plate transitions.

## 2. Results

### 2.1. Anatomy and Morphology

#### 2.1.1. *Begonia glabra* Aubl.

**Figure 1:**
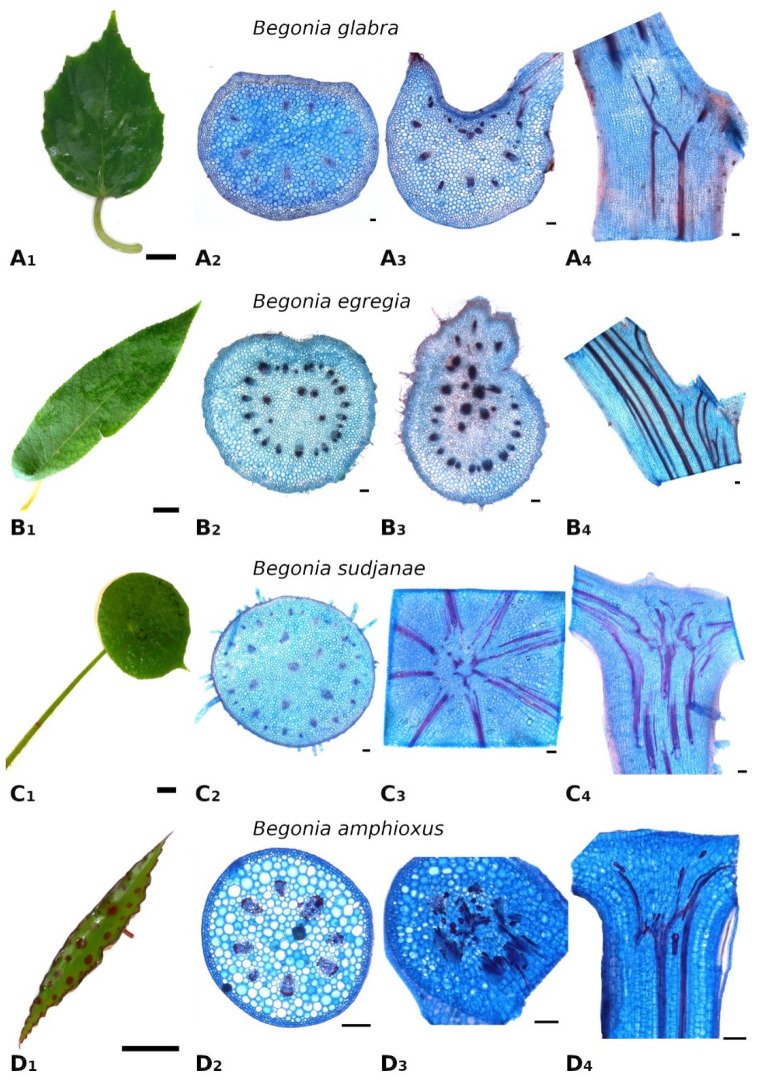
Leaf morphology and anatomy of *Begonia glabra* (**A**), *B. egregia* (**B**), *B. sudjanae* (**C**) and *B. amphioxus* (**D**). Leaf shape (A_1_), cross-section of petiole (A_2_), cross- (A_3_) and longitudinal (A_4_) section of petiole-lamina transition zone of *B. glabra.* Leaf shape (B_1_), cross-section of petiole (B_2_), cross- (B_3_) and longitudinal (B_4_) section of petiole-lamina transition zone of *B. egregia.* Leaf shape (C_1_), cross-section of petiole (C_2_), cross- (C_3_) and longitudinal (C_4_) section of petiole-lamina transition zone of *B. sudjanae.* Leaf shape (D_1_), cross-section of petiole (D_2_), cross- (D_3_) and longitudinal (D_4_) section of petiole-lamina transition zone of *B. amphixous.* Staining: astrablue/safranin. Scale: (1)—2 cm, (2–4)—100 µm. All samples were taken from one plant per species (two for *B. sudjanae*).

*Begonia glabra* is a perennial, climbing, non-peltate species distributed in Central and tropical South America [23]. The stems creep along the soil surfaces or climb attached to surrounding supports via adventitious roots [24]. The lamina is elliptic to ovate, nearly symmetrical (Figure 1(A_1_)) 3–11 cm wide and 5.5–17 cm long (width-to-length ratio: 1:1.7). The leaf thickness in intercostal areas is 0.4–0.7 mm. The leaf margin is slightly toothed. The petiole length ranges from 1–10 cm and 2–6.6 mm in width and has a D-shape cross section. All samples were taken from one plant.

**Figure 2:**
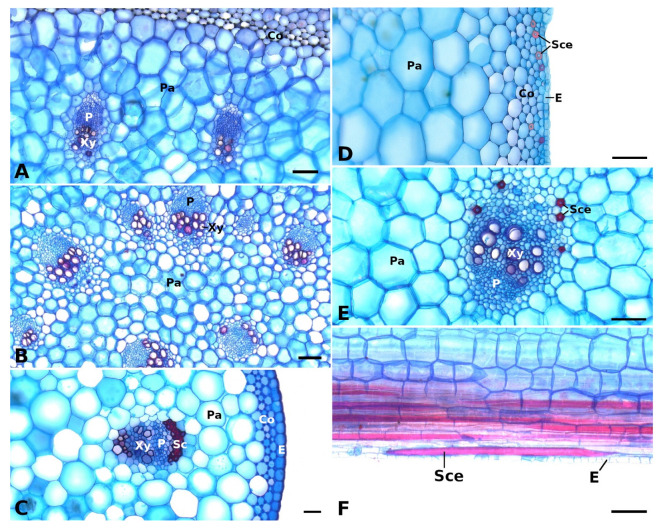
Vascular bundles and strengthening tissues in the petioles of *Begonia glabra* (**A**), *B. egregia* (**B**), *B. amphioxus* (**C**), and *B. sudjanae* (**D**–**F**). The cross sections (**A**–**E**) show vascular bundles with epidermis (E), phloem (P), and xylem (Xy) embedded in parenchyma (Pa), subepidermal collenchyma (Co) layers, sclerenchyma caps (Sc), and sclereid cells (Sce). In the petiole of *B. sudjanae* elongated sclereids cells (Sce) can be found (**F**, longitudinal section). Staining: astrablue/safranin, scale: 100 µm. All samples were taken from one plant per species (two for *B. sudjanae*).

**Figure 3:**
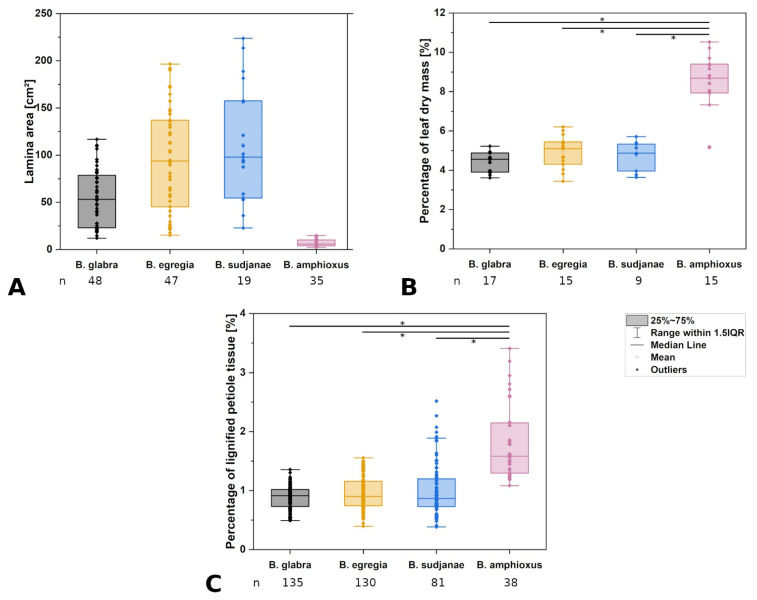
Comparison of leaf characters in different *Begonia* species. Lamina area in cm^2^ (**A**), percentage of leaf dry mass (**B**) and percentage of lignified tissue in the petiole (**C**) of *Begonia glabra*, *B. egregia*, *B. sudjanae,* and *B. amphioxus*. Percentage of dry mass refers to the percentage of the ratio between dry mass in mg to fresh mass in mg. Percentage of lignified petiole tissue refers to ratio of lignified tissue to non-lignified tissue in %. The box represents data between first and third quartile. Whiskers include data points within 1.5× of the interquartile range. The median is marked with a line, the mean with a square. Asterisks (*) mark significant differences between species analyzed via Kruskal–Wallis ANOVA. For each species, the sample size (n) is shown below the species name. *p*-values are shown in Table 2. All samples were taken from one plant per species (two for *B. sudjanae*).

The petiole of *B. glabra* (Figure 1(A_2_)) shows the same anatomical features across all leaf sizes. The adaxial side of the petiole is flattened to weakly concave, while the abaxial side is rounded resulting in the aforementioned D-shape. From base to apex of the petiole, the geometry of adaxial side of the petiole changes to a more concave geometry. Independent of leaf size, the cross-sectional petiole area at the base is significantly higher than at the apex. The cross section shows differently sized vascular bundles arranged in a peripheral ring. Independent of leaf size, the number of vascular bundles is approximately the same (10 to 12). The petiole also shows subepidermal layers of angular collenchyma tissue (Figure 2A). The only lignified tissue can be found in the xylem elements of the vascular bundles (Figure 2A). The percentage of lignified petiole tissue in *B. glabra* averages at 0.91% (IQR = 0.29%, n = 120, Figure 3C). When examining the lignified tissue from base to apex of the petiole independent of leaf size, a significant difference is apparent (*p* = 0.038) between apical and basal part of the latter which has slightly lower percentages of lignified tissue (0.86%, IQR = 0.25%, n = 45, see Supplementary Materials) than the apical part (0.97%, IQR= 0.25%, n = 45).

In the petiole-lamina transition zone of *B. glabra,* the abaxial vascular bundles continue from the petiole into the midrib of the lamina without branching (Figure 1(A_3_)). On the adaxial side, some of the vascular bundles branch out into the veins of the lamina in a simple branching pattern. In the longitudinal view, few cross-links between xylem fibers can be seen (Figure 1(A_4_)).

The midrib of the lamina is characterized by a larger vascular bundle on the abaxial side and several smaller bundles on the adaxial side embedded in parenchyma tissue (see Supplementary Materials). On both sides, layers of subepidermal collenchyma can be found. The intercostal area shows a few layers of parenchyma with large cells below adaxial and abaxial epidermis. Embedded between these parenchyma cells, the mesophyll is located.

The leaf fresh mass averages at 3.56 g (IQR = 2.98 g, n = 17) with a percentage of total leaf dry mass of 4.55% (IQR = 0.98%, n = 17, Figure 3B). Lowest percentage of dry mass can be found in the petiole (3.28%, IQR = 0.60%, n = 17). Percentage of venation and intercostal area dry mass are approximately the same at 4.59% (IQR = 0.79%, n = 17) and 4.65% (IQR = 1.04%, n = 17), respectively. Values are shown in Table 1.

**Table 1:** Median values of measured leaf variables of the different *Begonia* species. Interquartile range and sample size (n) are given in brackets. Leaf variables refer to measurements taken from the whole leaf (including petiole and lamina). Petiole and lamina variables show measurements of only petiole or lamina, respectively. All samples were taken from one plant per species (two for *B. sudjanae*).

		*B. glabra*	*B. egregia*	*B. sudjanae*	*B. amphioxus*
Leaf variables	Leaf fresh mass [g]	3.56 (2.98, n = 17)	5.80 (7.11, n = 15)	7.68 (11.22, n = 9)	0.22 (0.20, n = 15)
	Percentage of leaf dry mass [%]	4.55 (0.98, n = 17)	6.57 (1.45, n = 15)	4.87 (1.37, n = 9)	8.69 (1.46, n = 15)
Petiole variables	Petiole diameter [mm]	3.77 (1.97, n = 48)	4.56 (1.74, n = 47)	5.42 (2.14, n = 19)	1.12 (0.29, n = 30)
	Petiole length [mm]	41.21 (31.22, n = 48)	40.37 (23.45, n = 43)	200.18 (113.83, n = 19)	7.71 (13.51, n = 29)
	Petiole fresh mass [g]	0.28 (0.60, n = 17)	0.61 (0.57, n = 15)	4.88 (5.51, n = 9)	0.006 (0.017, n = 15)
	Percentage of petiole dry mass [%]	3.28 (0.60, n = 17)	5.10 (1.14, n = 15)	3.97 (0.73, n = 9)	8.69 (1.46, n = 15)
	Percentage of lignified petiole tissue [%]	0.91 (0.29, n = 120)	0.90 (0.42, n = 130)	0.87 (0.47, n = 81)	1.58 (0.85, n = 38)
	Tensile Young’s modulus of petiole [MPa]	14.72 (7.44, n = 21)	14.70 (5.17, n = 16)	20.79 (12.31, n = 39)	25.81 (8.78, n = 7)
	Flexural Young’s modulus of petiole [MPa]	50.12 (22.97, n = 10)	36.94 (30.54, n = 15)	48.80 (32.68, n = 46)	-
	Flexural rigidity of petiole [Nmm^2^]	428.70 (511.96, n = 10)	456.40 (1126.09, n = 15)	2351.98 (3237.60, n = 46)	-
Lamina variables	Lamina area [mm^2^]	5321.01 (5580.65, n = 48)	9371.22 (9187.16, n = 47)	9791.59 (10,306.53, n = 19)	581.00 (622.59, n = 35)
	Lamina thickness [mm]	0.50 (0.09, n = 16)	0.62 (0.07, n = 16)	0.39 (0.04, n = 9)	0.41 (0.07, n = 13)
	Venation fresh mass [g]	0.62 (0.49, n = 17)	1.57 (1.84, n = 15)	0.37 (0.64, n = 9)	0.026 (0.019, n = 15)
	Percentage of venation dry mass [%]	4.59 (0.79, n = 17)	6.52 (1.25, n = 15)	6.55 (2.87, n = 9	12.93 (3.56, n = 15)
	Structural Young’s modulus of venation [MPa]	27.90 (13.69, n = 84)	21.38 (9.74, n = 85)	23.94 (9.42, n = 40)	21.79 (13.29, n = 20)
	Tensile strength of venation [MPa]	1.94 (1.03, n = 84)	1.25 (0.41, n = 85)	2.90 (0.65, n = 40)	1.35 (0.42, n = 20)
	Intercostal area fresh mass [g]	2.27 (2.08, n = 17)	3.29 (4.57, n = 15)	2.81 (4.83, n = 9)	0.19 (0.17, n = 15)
	Percentage of intercostal area dry mass [%]	4.65 (1.04, n = 17)	6.87 (2.20, n = 15)	6.08 (2.00, n = 9)	10.62 (4.39, n = 15)
	Structural Young’s modulus of intercostal areas [MPa]	4.51 (5.87, n = 96)	4.68 (1.48, n = 87)	6.69 (2.23, n = 45)	8.46 (4.74, n = 23)
	Tensile strength of intercostal areas [MPa]	0.43 (0.22, n = 96)	0.27 (0.10, n = 87)	0.49 (0.13, n = 45)	0.78 (0.27, n = 23)

**Table 2:** *p*-values from Dunn’s tests after Kruskal–Wallis ANOVA. Associated plots with marked significant differences and sample sizes are shown in Figure 3 and Figure 4 (significance level is 0.05, significant values marked with *).

	Percentage of Leaf Dry Mass	Percentage of Lignified Petiole Tissue	Tensile Young’s Modulus of Petiole	Flexural Young’s Modulus of Petiole	Flexural Rigidity of Petiole	Young’s Modulus of Venation	Young’s Modulus of Intercostal Areas
*B. glabra—B. egregia*	0.82	1	1	0.23	1	* 1.71 × 10^-6^	0.11
*B. glabra—B. sudjanae*	1	1	* 9.23 × 10^−4^	1	* 0.005	1	0.006
*B. glabra—B. amphioxus*	* 7.55 × 10^−7^	* 1.18 × 10^−17^	* 3.00 × 10^−4^	-	-	* 0.01	* 7.71 × 10^−6^
*B. egregia—B. sudjanae*	1	1	* 0.01	* 0.03	8.79 × 10^−4^	* 0.02	* 1.50 × 10^−6^
*B. egregia—B. amphioxus*	* 0.001	* 1.52 × 10^−14^	* 0.001	-	-	1	* 1.87 × 10^−9^
*B. sudjanae—B. amphioxus*	* 0.003	* 1.25 × 10^−13^	0.42	-	-	0.37	0.24

**Figure 4:**
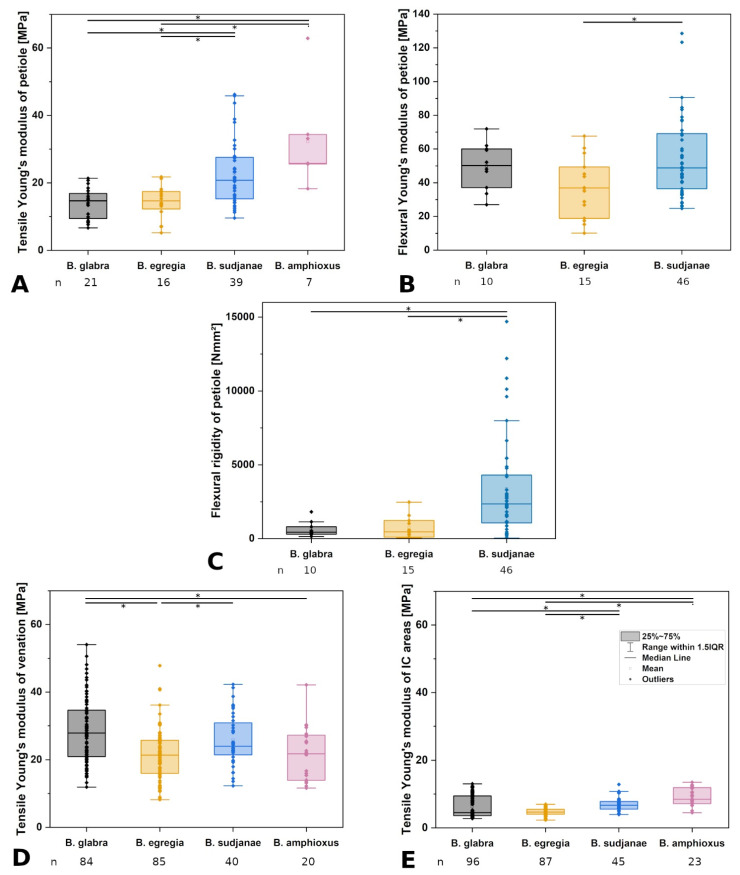
Comparison of mechanical leaf variables in different *Begonia* species. Tensile (**A**) and flexural (**B**) Young’s moduli of petiole in MPa, flexural rigidity of petiole (**C**) in Nmm^2^, tensile Young’s moduli of venation (**D**), and intercostal areas (**E**) in MPa of *Begonia glabra*, *B. egregia*, *B. sudjanae,* and *B. amphioxus*. The box represents data between first and third quartile. Whiskers include data points within 1.5× of the interquartile range. The median is marked with a line, the mean with a square. Asterisks (*) mark significant differences between species analyzed via Kruskal–Wallis ANOVA. For each species, the sample size (n) is shown below the species name. *p*-values are shown in Table 2. All samples were taken from one plant per species (two for *B. sudjanae*).

#### 2.1.2. *Begonia egregia* N.E.Br.

*Begonia egregia* is a perennial, caulescent species with peltate leaves with a petiole insertion close to the leaf margin and found only in Brazil [17,25]. The stem is woody at the base; young shoots are green and covered with hairs. The lamina is oblong, asymmetric, 3–9 cm wide and 9–32 cm long with an acute apex (Figure 1(B_1_)), width-to-length ratio: 1:3.3). In intercostal areas, the lamina is 0.4–0.8 mm thick. The pubescent petiole is 1–7.5 cm long, elliptic to circular in cross-section and 2.9–8.4 mm in diameter. All samples were taken from one plant.

In general, the petiole of *B. egregia* (Figure 1(B_2_)) exhibits a similar anatomy and geometry across all leaf sizes. At the base, the petiole is more elliptical in cross-section, with a deep indentation on the adaxial side. Toward the apex, the petiole geometry becomes circular and the indentation less prominent. The cross-sectional petiole area at the base is significantly higher than at the apex. The vascular bundles are arranged in a peripheral ring with some vascular bundles scattered in the center (Figure 1(B_2_)). The number of vascular bundles varies from 14 to 32. In the central vascular bundles, xylem elements are often not pointing toward the center (as in the outer ring), but surrounding the phloem or oriented outwards (Figure 2B). Several subepidermal layers of angular collenchyma are prominent. The percentage of lignified petiole tissue averages at 0.90% (IQR = 0.42%, n = 130, Figure 3C). With regard to the percentage of lignified petiolar tissue from base to apex, significant differences between basal and apical part are present (*p* = 1.35 × 10^−9^). The average percentage of lignified tissue increases from 0.77% (IQR = 0.17%, n = 45) at the base to 1.16% (IQR = 0.42%, n = 45) at the apex (see Supplementary Materials).

The petiole-lamina transition zone of *B. egregia* is different in geometry on the adaxial side, where some of the vascular bundles from the petiole branch into the lamina and some merge into larger bundles continuing into the midrib of the leaf (Figure 1(B_3_,B_4_)). The vascular bundles on the abaxial side run into the midrib of the lamina without conspicuous branching.

The anatomy of the midrib is relatively similar to the petiole anatomy (see Supplementary Materials). The vein is characterized by vascular bundles arranged in a ring and a few central bundles surrounded by parenchyma. On the abaxial side, a few layers of subepidermal collenchyma are prominent. The intercostal area shows a few layers of parenchyma with large cells below adaxial and abaxial epidermis. Between these parenchyma cells, the mesophyll is located.

The leaf fresh mass averages at 5.80 g (IQR = 7.11 g, n = 15). The petioles show the lowest percentage of dry mass (5.10%, IQR = 1.14%, n = 15), while venation and intercostal areas have slightly higher dry mass values (6.52%, IQR = 1.25% for venation, 6.87%, IQR = 2.20% for intercostal areas, n = 15). Values are shown in Table 1.

#### 2.1.3. *Begonia sudjanae* C.-A. Jansson

*Begonia sudjanae* is an acaulescent, rhizomatous, perennial herb with peltate leaves only known from Sumatra [17,23]. The lamina is ovate to orbicular, asymmetric, 5–15.5 cm wide, 5.5–20 cm long and pubescent (Figure 1(C_1_)), width-to-length ratio: 1:1.3). The lamina thickness in intercostal areas is 0.3–0.5 mm. The pubescent petiole is 7.5–34.5 cm long, circular in cross-section, and 2.4–8.4 mm in diameter. All samples were taken from two plants.

General geometry and anatomy of the petiole of *B. sudjanae* are similar across all leaf sizes. The petiole is circular in geometry from base to apex. The cross-sectional petiole area at the base is significantly higher than at the apex. The vascular bundles are arranged in two irregular peripheral rings, sometimes almost scattered (Figure 1(C_2_)). The vascular bundles of the outer ring are mostly smaller than the ones of the inner ring. The number of vascular bundles varies between 12 and 57 depending on petiole size. Several subepidermal layers of angular collenchyma are present as well as scattered sclereids (Figure 2D,E). These sclereids are elongated in shape (Figure 2F, approx. 300–1200 µm long) and located in the outer layers of collenchyma, often just below the epidermis or in close proximity to the xylem elements (Figure 2D,E). The amount of lignified petiolar tissue averages at 0.87% (IQR= 0.47%, n = 81, Figure 3C). When examining the lignified petiolar tissue from base to apex of the petiole, a significant difference between apical and basal part exists (*p* = 0.014), the latter of which has slightly lower percentages of lignified tissue (0.78%, IQR = 0.35%, n = 27, see Supplementary Materials) than the apical part (0.93%, IQR = 1.11%, n = 27).

A network of fibers characterizes the petiole-lamina transition zone of the peltate *B. sudjanae*. The xylem fibers from the petiole enter into the transition zone, branch, cross-link with each other and merge into larger fiber strands continuing into the veins of the lamina (Figure 1(C_3_,C_4_)). The aforementioned single elongated sclereid cells are also prominent in the transition zone.

The midrib of *B. sudjanae* is characterized by a larger vascular bundle on the adaxial side and smaller bundles toward the abaxial side surrounded by parenchyma (see Supplementary Materials). In association with these vascular bundles, single-cell sclereids are present. On abaxial and adaxial side, several layers of subepidermal collenchyma with scattered sclereids are prominent. The intercostal areas are defined by adaxial and abaxial epidermis with large cells with the mesophyll in between.

The leaf fresh mass of *B. sudjanae* averages at 7.68 g (IQR = 11.22 g, n = 9). The petiole shows the lowest dry mass averaging at 3.97% (IQR = 0.73%, n = 9), followed by the intercostal areas at 6.08% (IQR = 2.00%, n = 9) and venation at 6.55% (IQR = 2.87%, n = 9). The overall leaf dry mass averages at 4.87% (IQR = 1.37%, n = 9, Figure 3B). Median values are shown in Table 1.

#### 2.1.4. *Begonia amphioxus* Sands

*Begonia amphioxus* is a caulescent, peltate-leaved herb growing on limestone and limestone-associated substrates in Borneo [26]. The reddish stems are erect, glabrous, and lightly exceed 25 cm in height [26]. The peltate lamina is 0.8–2 cm wide, 4–12 cm long (width-to-length ratio: 1:5.4), narrowed to a point at both ends, green with red spots and glabrous (Figure 1(D_1_)). In intercostal areas, the lamina is 0.3–0.5 mm thick. The leaf margin is somewhat serrate. The short, reddish petiole attaches almost centrally to the lamina, is 0.5–2.5 mm long, circular in cross section, and 0.5–1.5 mm in diameter. All samples were taken from one plant.

The petiole of *B. amphioxus* (Figure 1(D_2_)) shows the same general anatomical features across all leaf sizes. The vascular bundles are arranged in a peripheral ring. Their number varies between 5 and 8. The vascular bundles are characterized by lignified xylem elements. In some larger leaves, sclerenchyma caps associated with the vascular bundles and few lignified cells in close proximity to the xylem are present (Figure 2C). The petiole also shows several layers of subepidermal angular collenchyma (Figure 2C). The percentage of lignified petiolar tissue averages at 1.58% (IQR = 0.85%, n = 38, Figure 3C). From the basal part of the petiole to the apex, the percentage of lignified tissue slightly increases (from 1.37%, IQR = 1.62% to 1.86%, IQR = 0.99%, n = 9, see Supplementary Materials), although not significantly (*p* = 0.73).

The xylem fibers from the petiole branch in the petiole-lamina transition zone, and some crosslink with each other before running into the main veins of the lamina (Figure 1(D_3_,D_4_)). Additionally, single sclereids scattered between vascular bundles are present in the transition zone.

The midrib of *B. amphioxus* is composed of a single large vascular bundle. A few collenchyma cells below the abaxial epidermis are associated with this vascular bundle. In the intercostal areas, the adaxial epidermis is characterized by large cells while the abaxial epidermis is smaller in cell size. The mesophyll is embedded between adaxial and abaxial epidermis (see Supplementary Materials).

The leaf fresh mass averages at 0.22 g (IQR = 0.20 g, n = 15). The petiole shows the lowest dry mass (8.69%, IQR = 1.46%, n = 15), followed by intercostal areas (10.62%, IQR = 4.39%, n = 15) and venation (12.93%, IQR = 3.56%, n = 15). Median values are shown in Table 1.

### 2.2. Biomechanics

#### 2.2.1. *Begonia glabra* Aubl.

Tensile testing of the different structural elements of *B. glabra* revealed low structural Young’s moduli for intercostal areas (4.51 MPa, IQR = 5.87 MPa, n = 96, Figure 4E), values twice as high for the petiole (14.72 MPa, IQR = 7.44 MPa, n = 21, Figure 4A) and the highest values for the venation (27.90 MPa, IQR = 13.69 MPa, n = 84, Figure 4D). The venation shows higher tensile strength than the intercostal areas averaging at 1.94 MPa (IQR = 1.03 MPa, n = 84) and 0.43 MPa (IQR = 0.22 MPa, n = 96, Table 1), respectively.

Three-point bending tests of the petiole of *B. glabra* show higher values for the Young’s modulus (50.12 MPa, IQR = 22.97 MPa, n = 10, Figure 4B) compared to tensile testing. The flexural rigidity averages at 428.70 Nmm^2^ (IQR = 511.96 Nmm^2^, n = 10, Figure 4C).

Median values are shown in Table 1. All samples were taken from one plant.

#### 2.2.2. *Begonia egregia* N.E.Br.

Tensile testing revealed the highest Young’s moduli for the venation (21.38 MPa, IQR = 9.74 MPa, n = 85, Figure 4D), followed by the petiole (14.70 MPa, IQR = 5.17 MPa, n = 16, Figure 4A) and intercostal areas (4.68 MPa, IQR = 1.48 MPa, n = 87, Figure 4E). Tensile strength of the veins is higher than values of intercostal areas with 1.25 MPa (IQR = 0.41 MPa, n = 85) and 0.27 MPa (IQR = 0.10 MPa, n = 87), respectively.

Three-point bending tests of the petioles of *B. egregia* revealed higher values for the Young’s modulus (36.94 MPa, IQR = 30.54 MPa, n = 15, Figure 4B) in comparison to tensile testing. The flexural rigidity of the petioles averages at 456.40 Nmm^2^ (IQR = 1126.09 Nmm^2^, n = 15, Figure 4C).

Median values are shown in Table 1. All samples were taken from one plant.

#### 2.2.3. *Begonia sudjanae* C.-A. Jansson

Tensile tests of the structural elements of *B. sudjanae* revealed the highest *E* values for the venation (23.94 MPa, IQR = 9.42 MPa, n = 40, Figure 4D). Respective values for the petiole are 20.79 MPa (IQR = 12.31 MPa, n = 39, Figure 4A), followed by intercostal areas (6.69 MPa, IQR = 2.23 MPa, n = 45, Figure 4E). The venation shows higher tensile strength than the intercostal areas averaging at 2.90 MPa (IQR = 0.65 MPa, n = 40) and 0.49 MPa (IQR = 0.13 MPa, n = 45), respectively.

Three-point bending tests of the petioles of *B. sudjanae* show higher Young’s modulus values compared to tensile tests averaging at 48.80 MPa (IQR = 32.68 MPa, n = 46, Figure 4B). Basal petiole samples show lowest values for flexural Young’s modulus (40.15 MPa, IQR = 12.82 MPa, n = 15), followed by middle (55.34 MPa, IQR = 29.05 MPa, n = 16) and apical petiole samples (68.21 MPa, IQR = 36.60 MPa, n = 15, see Supplementary Materials). From basal to apical part of the petiole, the flexural Young’s modulus increases significantly. Kruskal-Wallis ANOVA and Dunn’s test revealed significant differences between basal and middle part (*p* = 0.02), as well as basal and apical part of the petiole (*p* = 0.009). The flexural rigidity of the petiole averages at 2351.98 (IQR = 3237.60, n = 46, Figure 4C). Basal petiole samples show highest values for flexural rigidity (3300.68 Nmm^2^, IQR = 3239.43 Nmm^2^, n = 15), followed by middle (2769.74 Nmm^2^, IQR = 4328.43 Nmm^2^, n = 16) and apical petiole samples (1068.34 Nmm^2^, IQR = 1937.23 Nmm^2^, n = 15, see Supplementary Materials). From basal to apical part of the petiole, the flexural rigidity decreases significantly. Kruskal–Wallis ANOVA and Dunn’s test revealed significant differences between basal and apical part (*p* = 0.007), as well as middle and apical part of the petiole (*p* = 0.01).

All median values are shown in Table 1. All samples were taken from two plants.

#### 2.2.4. *Begonia amphioxus* Sands

Tensile tests of the structural elements show highest *E* values for the petiole (25.81 MPa, IQR = 8.78 MPa, n = 7, Figure 4A), followed by venation (21.79 MPa, IQR = 13.29 MPa, n = 20, Figure 4D) and lowest for intercostal areas (8.46 MPa, IQR = 4.74 MPa, n = 23, Figure 4E). Tensile strength is higher in venation (1.35 MPa, IQR = 0.42 MPa, n = 20) than in intercostal areas (0.78 MPa, IQR = 0.27 MPa, n = 23).

All median values are shown in Table 1. All samples were taken from one plant.

## 3. Discussion

As one of the largest genera of the flowering plants, *Begonia* shows a huge diversity in growth forms, sizes, and leaf geometries [17]. The four *Begonia* species analyzed in this comparative case study represent only a small fraction of this diversity and differ in growth habit (climbing, shrub-like, herbaceous), leaf size (few cm^2^ to over 200 cm^2^ lamina area, Figure 3A), and geometry. When comparing the cross-sectional geometry of the petiole, two groups are apparent. While non-peltate *B. glabra* and eccentric peltate *B. egregia* are characterized by a mirror-symmetrical petiole cross-section, the more central peltate *B. sudjanae* and *B. amphioxus* show radially symmetrical, circular petioles. The circular petiole geometry can also be found in other peltate Begonias, e.g., *B. nelumbiifolia* and *B. peltata* [11]. The shape and geometry of the petiole affect the mechanical properties, more precisely the resistance to bending and twisting and thus, the resistance to damage. The leaves of *B. glabra* and *B egregia* are horizontally oriented. A D-shaped cross section (*B. glabra*) or an elliptical cross section with an indentation (*B. egregia*) are more resistant to downward bending while being highly flexible in torsion and thus, are of advantage for leaves with a 2D configuration [27,28]. In contrast, in leaves with a 3D configuration as in *B. sudjanae* and *B. amphioxus* the petiole must be resistant to bending and torsion in all directions. Thus, a circular petiole cross section is beneficial as it does not have a preferred direction for bending. Regarding anatomy, petioles of all four species show the characteristic tissue arrangement of *Begonia* [19]. The vascular bundles are embedded in parenchymatous tissue and either are arranged in one ring (like in *B. glabra* and *B. amphioxus*, Figure 1(A_2_,D_2_)), sometimes with additional scattered central bundles (*B. egregia*, Figure 1B_2_) or in more rings that can appear almost scattered (*B. sudjanae*, Figure 1(C_2_)). The subepidermal collenchyma present in all four studied species, is known for *Begonia* petioles and generally common as reinforcement in petioles [5,8]. In two species, additional strengthening tissue was found. The elongated sclereids in *B. sudjanae* that are located in the periphery of the petiole or associated with the vascular bundles (Figure 2D–F) are known from several *Begonia* species [18,19]. In close association with the vascular bundles, sclereids are also found in the lamina providing additional stiffness to the leaf [19]. The sclerenchyma caps (Figure 2C) found in some large leaves of *B. amphioxus* are another way the *Begonia* petiole is mechanically strengthened. These fibrous caps are known from *Begonia* and additionally, sclerenchymatous caps as stiffening elements are common in petioles of non-peltate and peltate leaves [11,13,18]. With the cross-sectional petiole geometry and arrangement of peripheral collenchyma, xylem fibers and in some species, sclerenchyma, the petioles of the four *Begonia* species are optimally designed to provide the required stiffness to secure their function while being flexible enough to prevent damage by the various mechanical stresses.

The high amount of hydrostatic tissue, like parenchyma and collenchyma, visible in the petiole and lamina cross-sections is reflected in the low percentage of leaf dry mass and percentage of lignified tissue in three out of four species (Table 1, Figure 3B,C). Not surprisingly, these low amounts of lignified tissue point toward turgor pressure as crucial for the stability of the leaf. In hydrostatic tissues, such as parenchyma and collenchyma representing more than 98% of petiole tissue of the studied species, turgor pressure defines the mechanical properties of the whole structure [3,4]. At full turgor, external stresses are redistributed across the cell walls which results in stiffening of the tissue as a whole [4]. When deprived of water, *E* values of parenchyma and collenchyma decrease and show a higher flexibility and lower flexural rigidity of the petiole [29]. The impact of the above-mentioned fiber bundles in the plant individual of *B. amphioxus* now becomes apparent as this species shows higher percentages of leaf dry mass and lignified petiolar tissue than the other analyzed species. In comparison to other *Begonia* species, the dry mass values correspond well with measured dry masses from *B. peltata* and *B. nelumbiifolia* [11]. In contrast, the amount of lignified tissue measured in this study is lower than the values in the study of Wunnenberg et al. [11] who measured between 4 and 8% of lignified strengthening tissue (in comparison to 0.9 to 1.8% in this case study). This difference might be explained by the low sample size in Wunnenberg et al. [11], but it must also be considered that these few analyzed plants of four species represent only a small fraction of the large genus of *Begonia*. Thus, variations from species to species in amount of lignified tissue are most likely.

Comparing the petiole-lamina transition zones in this case study of the four species, two groups are apparent. While both species with marginal and eccentric petiole insertion can be classified into the simple branching type, the two species with a more central petiole insertion can be sorted into the net-like structure type (according to Wunnenberg et al. [11]). This net-like structure was also detected in other *Begonia* species with a more central petiole insertion [11] indicating a change and increase in complexity in fiber orientation and branching from non-peltate- to peltate-leaved species. Especially the intermediate leaf shape of *B. egregia* with a slightly more complex transition zone than the non-peltate *B. glabra* seems to support this hypothesis (Figure 1A,B). In non-peltate leaves the vascular bundles mostly run in one direction into the lamina, while in peltate leaves, the bundles run radially in several directions. Therefore, the increase of complexity in the transition zone could simply be explained by the change of spatial configuration (from 2D to 3D) and the need of vascular bundles sustaining all parts of the lamina. Nonetheless, the organization of the petiole-lamina transition zone plays an important role in load-dissipation and, as a damage-resistant structure, is optimized to secure the connection between petiole and lamina [21,22]. While the mechanical testing of only the transition zone is challenging, there are several approaches for tensile and torsion tests, as well as deformation tests that could shed further light onto the mechanical properties of the petiole-lamina transition zone [21,22].

Comparing the mechanical properties, all analyzed species of this case study show tensile *E* values in a similar range in venation (21.38 MPa to 27.90 MPa, Table 1, Figure 4D) and intercostal areas (4.51 to 8.48 MPa, Table 1, Figure 4E), respectively. The intercostal areas mostly comprise of upper and lower epidermis and a parenchymatous mesophyll with large air spaces in between. Additionally, parenchyma layers or an epidermis with large cells are prominent in all four species. The mechanical support of parenchyma depends on turgor and packaging density [4]. Densely packed parenchyma cells (e.g., in petioles) provide much more mechanical support than the spongy mesophyll found in the intercostal areas of the leaf. Thus, lower *E* values are expected. In contrast, the venation is composed of vascular tissue including the lignified xylem elements surrounded by parenchyma and, in some species, collenchyma, and is a critical component in ensuring the stiffness of the lamina [1,4,30]. Thus, to support the lamina, higher *E* values in the scaffolding (venation) are advantageous. Whereas tensile *E* values in intercostal areas and venation are similar across the four studied species, differences in the petioles are apparent (Table 1, Figure 4A). In non-peltate *B. glabra* and eccentric peltate *B. egregia*, tensile *E* averages at 14.72 and 14.70 MPa, respectively. The higher *E* values in *B. sudjanae* (20.79 MPa) and *B. amphioxus* (25.81 MPa) can be attributed to the additional strengthening tissues in the petioles. Especially lignified tissue such as the single cell sclereids and sclerenchyma strands increase stiffness significantly resulting in higher Young’s moduli [3,4]. The impact of the amount of lignified tissue in the petiole can be seen in the simultaneous increase of lignified petiolar tissue and Young’s modulus (Figure 3C and Figure 4A). In comparison to tensile testing, the values for flexural Young’s modulus are higher in all three tested species (Figure 4A,B, Table 1). While under tensile loads only the amount of strengthening tissues affect the petiole stiffness, under bending loads the stiffness is additionally affected by the position of these strengthening tissues within the petiole cross-section resulting in higher flexural *E* values. The quite similar *E* values across all three species (*B. glabra*: 50.12 MPa, *B. egregia*: 39.94 MPa and *B. sudjanae*: 48.80 MPa) could be explained by the similar arrangement of xylem fibers (in one or two rings, Figure 1) and collenchyma (below the epidermis) in all species analyzed in this case study resulting in a similar stiffness. The lower tensile *E* values might also be explained by the difficulty of sufficiently clamping the petiole samples in tensile tests without damaging them. Thus, the softer polyurethane layer on the clamps could influence the measurements and slippage of the samples might occur. When comparing the flexural rigidity of the petioles, *B. sudjanae* shows significant higher values (2351.98 Nmm^2^, Figure 4C) than *B. glabra* (428.70 Nmm^2^) and *B. egregia* (456.40 Nmm^2^). This difference can be attributed to the higher petiole diameters and second moments of area in *B. sudjanae* in general and especially in basal parts of the petiole. To fully characterize and compare the biomechanical properties of the *Begonia,* petioles compression and torsional testing are required.

The percentage of lignified tissue in the petiole cross-section increases significantly in most species analyzed in this case study from base to apex. Since the types of stresses that affect the petiole are different depending on the part of the petiole and since tissue composition and arrangement define the mechanical properties, differences in anatomy are expected [3,4]. At the base of the petiole the highest bending stresses occur, whereas the apex is exposed to high torsional shear stresses [3]. The parenchyma in the center of *Begonia* petioles can deal with shear stresses from bending of the petiole. The subepidermal collenchyma layers can deal with torsional shear stresses [3]. In contrast, sclerenchymatous tissue below the epidermis (vascular bundles or sclereids/sclerenchyma caps in *Begonia*) stiffens the petiole. Since twisting and bending stresses are especially high at the base of the petiole, a high proportion of parenchyma and collenchyma is optimal, whereas at the apex, higher amounts of lignified tissue additionally help to cope with higher torsional shear stresses [3]. These tissue proportions and arrangements result in higher flexural rigidity and lower flexural Young’s modulus at the base of the petiole, where the highest bending stresses occur, as can be seen in *B. sudjanae*, and lower flexural rigidity and higher Young’s modulus in the apical part of the petiole.

## 4. Materials and Methods

### 4.1. Plant Material

Samples of *Begonia glabra* Aubl., *B. egregia* N.E.Br., *B. sudjanae* C.-A. Jansson and *B. amphioxus* Sands were provided by the Botanical Garden of Technische Universität Dresden, Germany. All plants were cultivated in the greenhouse under tropical conditions (20 ± 5 °C, humidity: 70%, no artificial lighting). The selection of the plant species included one non-peltate leaved species (*B. glabra*), one peltate species with the petiole insertion point close to the leaf margin (*B. egregia*), and two peltate species with a more central petiole insertion point (*B. sudjanae* and *B. amphioxus*). One plant individual per species (two for *B. sudjanae*) was available for sampling. Before sampling, lamina lengths were measured. Only intact, undamaged leaves were taken and collected between March 2021 and March 2022. Young and growing leaves were excluded from sampling but no detailed maturity analysis was conducted. For caulescent species, no samples were taken from the first two to three leaves/pairs of leaves of a growing shoot. For the acaulescent species *B. sudjanae*, samples were taken only by visual estimate. Young, growing leaves of *B. sudjanae* showed lighter leaf color, denser pubescence, and often a not fully unfolded lamina in comparison to adult leaves. In total, 58 leaves of *B. glabra*, 61 leaves of *B. egregia*, 34 leaves of *B. sudjanae,* and 35 leaves of *B. amphioxus* were sampled. Fresh leaves were transported in airtight containers and scanned (Ricoh MP C3004ex, Ricoh Company, Ltd., Chuo, Tokyo, Japan) or photographed (Lumix DMC-G81, Panasonic, Kodama, Osaka, Japan) from both sides and subsequently processed within a few hours or preserved in ethanol (70%) for later analysis.

### 4.2. Anatomy and Morphology

Leaf and petiole dimensions for all samples (taken from one to two plants per species) were measured based on scans or photographs using the software ImageJ (National Institutes of Health, Bethesda, MD, USA). Lamina length was determined at the longest point along the midrib, leaf width perpendicular to the midrib at the widest part. Compilation of measured variables was made in MS Excel (Microsoft Corporation, Redmont, WA, USA).

For the measurement of fresh and dry weight, petioles, venation, and intercostal areas of nine to 15 leaves of different sizes were separated using a scalpel or razor blade. To minimize water loss during cutting, the leaves were stored in water. Fresh and dry weights of the structural elements were measured with a precision scale (Mettler Toledo XA205DU, Columbus, OH, USA). To determine the dry weight, the fresh leaf samples were dried to constant mass at 60 °C in a drying cabinet (Heraeus T12, Heraeus Instruments, Hanau, Germany).

**Figure 5:**
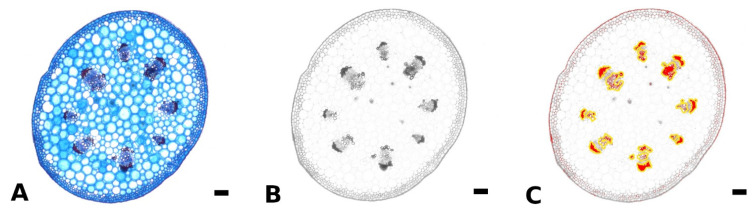
Method for measurement of lignified petiole tissue using ImageJ. The original colored image of a petiole cross section of *Begonia amphioxus* (**A**) was split into RGB channels, and only the blue channel (where lignified tissue appears dark) was used for further analysis (**B**). Via the threshold function, the lignified tissue was highlighted (red) and subsequently, added to Regions of Interest (yellow outline), edited and measured (**C**). Scale: 100 µm.

Fresh, non-embedded cross sections of the petiole, midrib, and intercostal area and cross and longitudinal sections of the petiole-lamina transition zone were prepared with a razor blade or vibratome (Hyrax V50, Carl Zeiss AG, Jena, Germany). Petiole cross sections were taken from basal, central, and apical parts of the petioles of differently sized leaves. Sections were bleached in sodium hypochlorite solution (2.8%), washed in water, stained with astrablue (1% in 2% acetic acid)/safranin (1% in H_2_O) and differentiated in ethanol (70%). Astrablue stains non-lignified tissue blue, safranin stains lignified tissue red. The stained sections were photographed using a light microscope (VHX-970F, Keyence AG, Osaka, Japan) and integrated camera (VHX-970F, CMOS-image sensor, Keyence AG, Osaka, Japan). The amount of lignified strengthening tissue in the petiole was measured for at least nine leaves per species. For each of these leaves, three to five sections from each basal, central, and apical part of the petiole were analyzed using the software ImageJ (National Institutes of Health, Bethesda, MD, USA). First, the area of the cross section was measured using the binary function. For the area measurement of the lignified tissue, the colored images were split into RGB channels and the blue channel was used for further analysis (Figure 5A,B). In the blue channel image, red/pink stained tissues appear dark and blue stained tissues appear light. With the threshold function all darker areas up to a certain value could be highlighted. These highlighted areas could either be measured directly via the measurement function (setting the measurement to “limit to threshold” in the settings), or were added to Regions of Interest. Subsequently, these Regions of Interest could be edited with the brush selection tool, if needed, combined (Figure 5C) in the ROI manager and measured via the measurement function.

### 4.3. Mechanical Testing

Individual samples (taken from one to two plants per species) of petiole, venation and intercostal areas were subjected to tensile tests until fracture using a universal testing machine (zwickiLine, Zwick/Roell GmbH &Co. KG, Ulm, Germany) and corresponding software (testXpert II V3.5, Zwick/Roell GmbH &Co. KG, Ulm, Germany). Additionally, samples of petioles were subjected to three-point bending tests using a universal testing machine (Zwick/Roell AllroundLine Z005, Zwick/Roell GmbH &Co. KG, Ulm, Germany) and corresponding software (see above). Due to small petiole dimensions of *B. amphioxus*, only tensile tests were performed for this species. Samples of nine to fifteen leaves per testing method were prepared depending on the leaf size and tested. For tensile tests, petioles were cut into pieces of 10 to 40 mm. The venation was separated from intercostal areas using a razor blade and cut to a length of 10 to 40 mm. The intercostal areas were cut into samples with a width-to-length-ratio of 1:8. For three-point bending tests, petioles were tests as a whole or cut into pieces of at least 30 mm depending on the length of the petiole. Width of petiole and venation samples and thickness of intercostal area samples were measured using a digital caliper (Precise PS 7215, Burg-Wächter, Wetter-Volmarstein, Germany). Where needed, samples were fixed onto small pieces of paper using instant glue (UHU instant glue, UHU GmbH & Co. KG, Bühl, Germany) to prevent slippage of the samples during tensile testing. Approx. five samples of petiole (if possible), venation and intercostal areas per leaf were tested. To minimize water loss, the samples were stored between wet paper sheets until testing. For tensile tests, clamping length was set to 5 mm, 10 mm or 20 mm, respectively, depending on sample dimensions. Clamps with a thin layer of polyurethane were used. For three-point bending tests, supporting width was set to 20 to 50 mm. Pre-load was set to 0.01 N and testing speed to 2 mm/min. Resulting forces were recorded using a 50 N and 5 kN load cell (50 N: Type: KAP-Z, AST Angewandte Systemtechnik GmbH, Dresden, Germany, 5 kN: Zwick/Roell xforce P, Zwick/Roell GmbH & Co. KG, Ulm, Germany). The software OriginLab 2021 Pro (OriginLab Corporation, Northhampton, MA, USA) was used for further analysis. For tensile tests, stress–strain diagrams were created from measured force and displacement. Where possible, for each sample the structural Young’s modulus (*E*) was determined from the linear elastic part of the stress–strain curve and the tensile strength determined as the maximum stress. For three-point bending, the Young’s modulus (*E*) was calculated using the following formula:

(1) $$E=b\cdot \frac{l^{3}}{48\cdot I}$$

with *b* being the slope of the displacement-force diagram, *l* the supporting width, and *I* the second moment of area. Flexural rigidity (*EI*) was calculated multiplying Young’s modulus by second moment of area.

### 4.4. Statistical Analysis

The statistical analysis of data was performed with OriginLab 2021 Pro (OriginLab Corporation, Northhampton, MA, USA). To check for normal distribution of the data, the Shapiro–Wilk test was used. Samples from one species were considered dependent, as leaf samples were taken mostly from one plant. In the species comparison, samples were considered independent. All data are given as median and interquartile range (IQR) with respective sample size (n). The Student’s t-test for paired samples and the Wilcoxon signed-rank test were applied to test the significance of possible differences between percentages of lignified tissue in apical and basal parts of the petiole for normally distributed and non-normally distributed data, respectively. For species comparison and comparison of biomechanical variables in relation to sample position, the Kruskal–Wallis ANOVA with Dunn’s test were used. For all tests, a significance level of 0.05 was chosen.

## 5. Conclusions

In this comparative case study, we provided an analysis of morphology, anatomy, and biomechanics of the leaves of four *Begonia* species with different leaf sizes, geometries, and petiole attachment points to understand the principles behind tensile and bending stiffness, tensile fracture mechanics, and damage prevention of the leaf.

The comparison between the four *Begonia* species highlights the interaction between the different tissues in the leaves and the connection between leaf shape, anatomy, and biomechanical properties allowing the leaf to resist external stresses and prevent damage. Due to the 3D architecture of the peltate leaves in comparison to non-peltate leaves, a different organization of the petiole-lamina transition zone is needed to withstand the 3-dimensional and rotational loads. This difference in organization can be seen in the *Begonia* species described in this study. Since the petiole-lamina transition zone in peltate leaves is able to dissipate the loads that affect the lamina, damage from these loads is much more likely to occur within the petiole or the lamina, but not in the transition zone itself. Due to the abilities of load dissipation and damage control, the analysis of the transition zone will be especially promising for possible biomimetic adaptations.

## Data Availability

Data are contained within the article or Supplementary Materials.

## Supplementary Materials

The following are available online at https://www.mdpi.com/article/10.3390/plants11233297/s1, Document S1: Staining workflow for fresh, non-embedded sections. Figure S1: Percentage of lignified petiole tissue of *B. glabra* at the basal, central and apical part of the petiole. Figure S2: Transversal anatomy of venation and intercostal areas of *Begonia*. Venation (A) and intercostal area (B) of *B. glabra*, venation (C) and intercostal area (D) of *B. egregia*, venation (E) and intercostal area (F) of *B. sudjanae* and venation and intercostal area (G) of *B. amphioxus*. Adaxial side—top, scale: 100 µm. Figure S3: Percentage of lignified petiole tissue of *B. egregia* at the basal, central and apical part of the petiole. Figure S4: Percentage of lignified petiole tissue of *B. sudjanae* at the basal, central and apical part of the petiole. Figure S5: Percentage of lignified petiole tissue of *B. amphioxus* at the basal, central, and apical part of the petiole. Figure S6: Flexural Young's modulus in MPa of *B. sudjanae* at the basal, central and apical part of the petiole. Figure S7: Flexural rigidity in Nmm^2^ of *B. sudjanae* at the basal, central and apical part of the petiole. Table S1: Values of morphological, anatomical, and biomechanical variables.
