# Supplementary material for: Anatomy and Biomechanics of Peltate Begonia Leaves—Comparative Case Studies
Source: Plants (Basel). 2022 Nov 29;11(23):3297. doi: 10.3390/plants11233297 (PMC9738572; doi:10.3390/plants11233297)
Supplement: Supplementary file 1 [file plants-11-03297-s001.zip › Document S1.pdf]

Staining workflow for fresh, non-embedded sections

Fresh, non-embedded cross sections of the petiole and cross and longitudinal sections of the petiole-lamina transition zone were prepared with a razor blade or vibratome (Hyrax V50, Carl Zeiss AG, Jena, Germany). Sections were bleached in sodium hypochlorite solution (2.8 %) for 8-10 min, washed in water for at least 10 min, stained with astrablue (1 %) / safranin (1 %) for 1 min, differentiated in ethanol (70 %) for 1 min and afterwards stored in water until microscopic analysis.
