# Supplementary material for: Anatomy and Biomechanics of Peltate Begonia Leaves—Comparative Case Studies
Source: Plants (Basel). 2022 Nov 29;11(23):3297. doi: 10.3390/plants11233297 (PMC9738572; doi:10.3390/plants11233297)
Supplement: Supplementary file 1 [file plants-11-03297-s001.zip › plants-1995998-SM Figures.pdf]

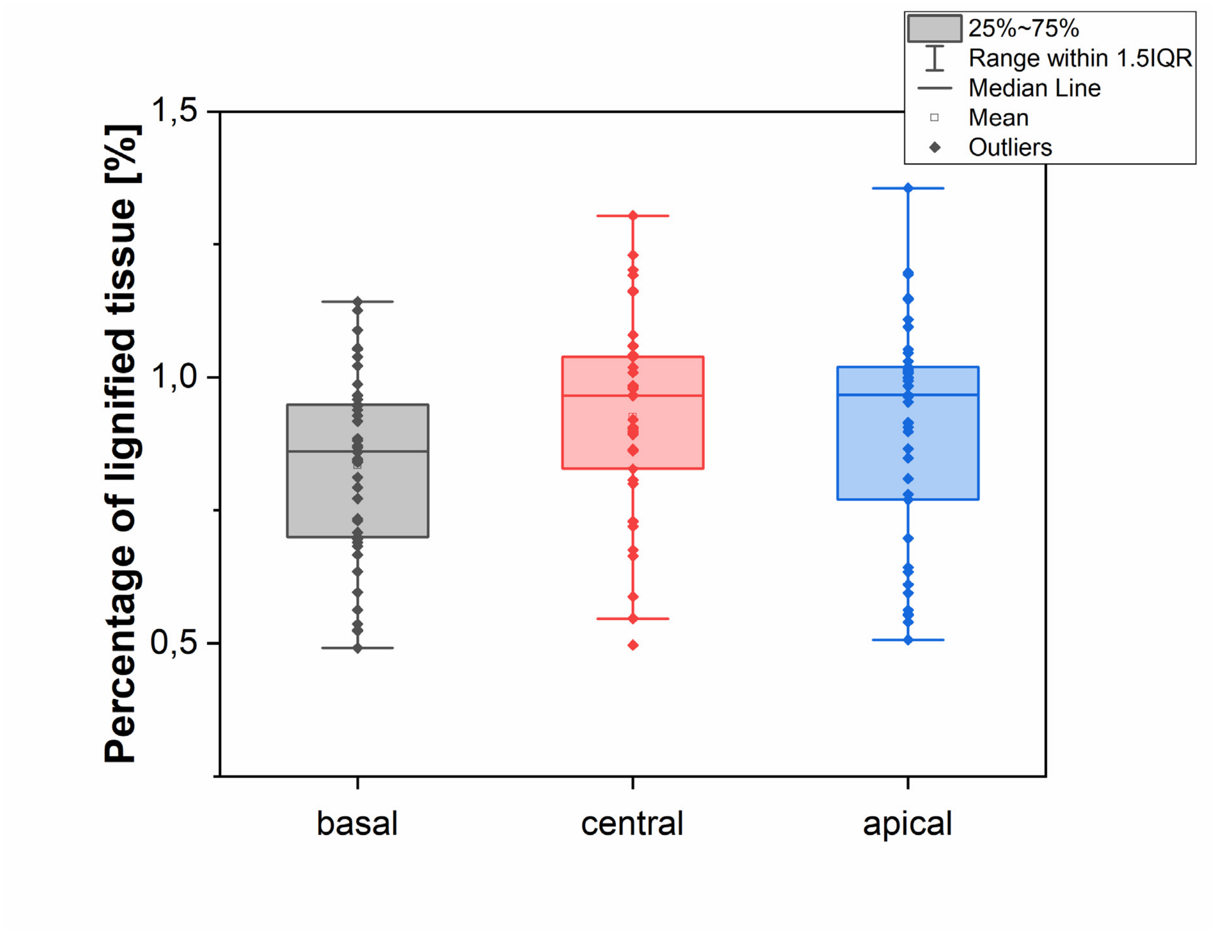

Figure S1: Percentage of lignified petiole tissue of *B. glabra* at the basal, central and apical part of the petiole.

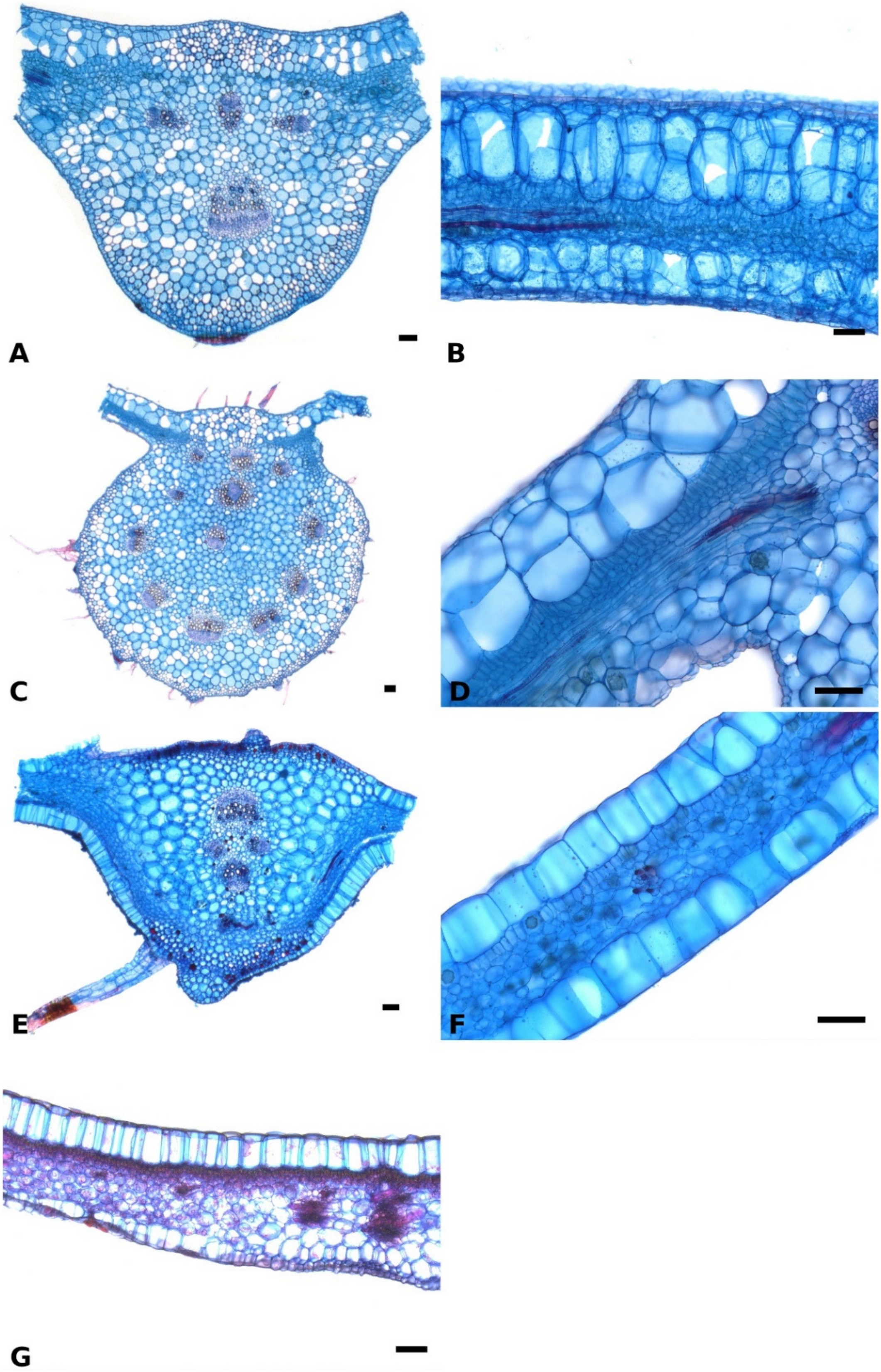

Figure S2: Transversal anatomy of venation and intercostal areas of *Begonia*. Venation (A) and intercostal area (B) of *B. glabra*, venation (C) and intercostal area (D) of *B. egregia*, venation (E) and intercostal area (F) of *B. sudjanae* and venation and intercostal area (G) of *B. amphioxus*. Adaxial side—top, scale: 100 μm.

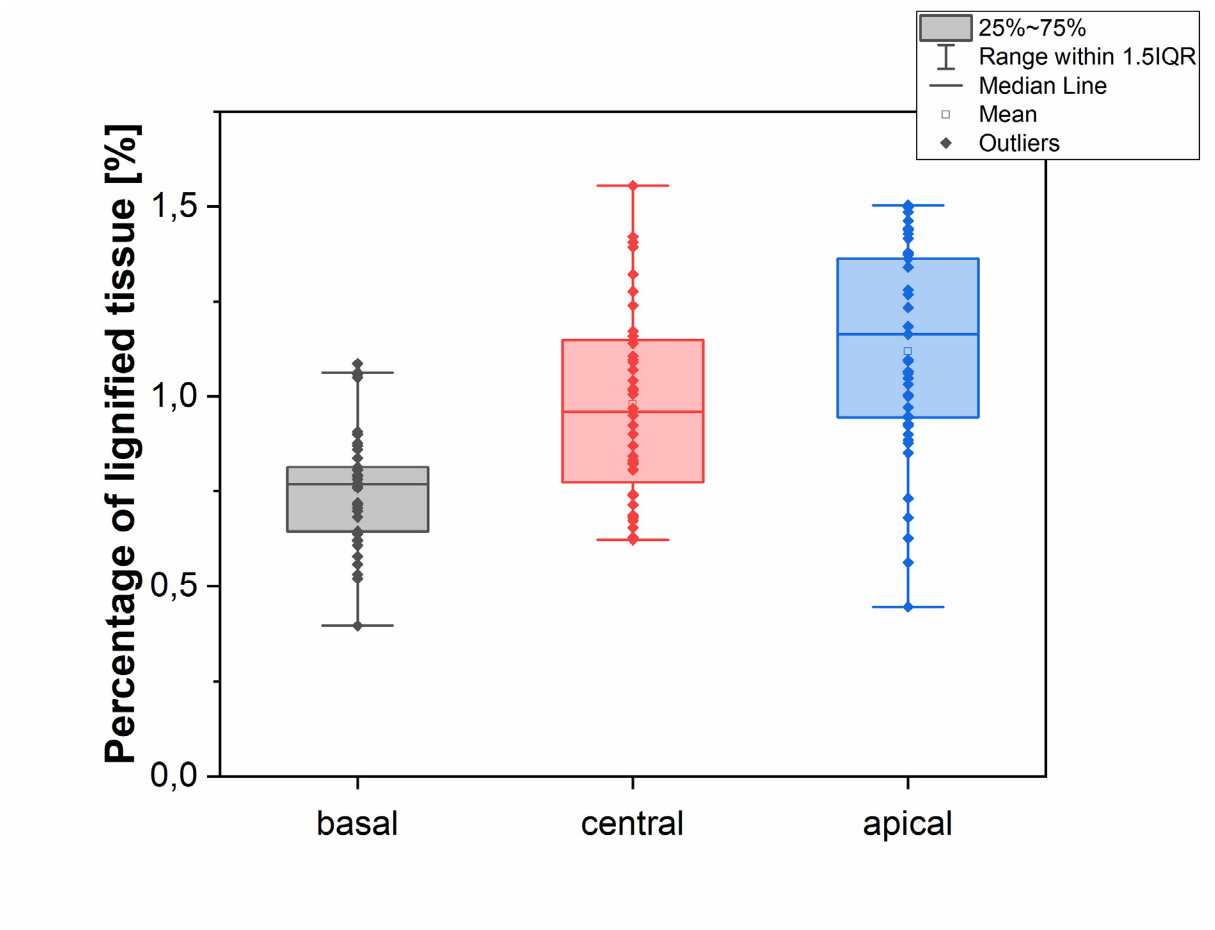

Figure S3: Percentage of lignified petiole tissue of *B. egregia* at the basal, central and apical part of the petiole.

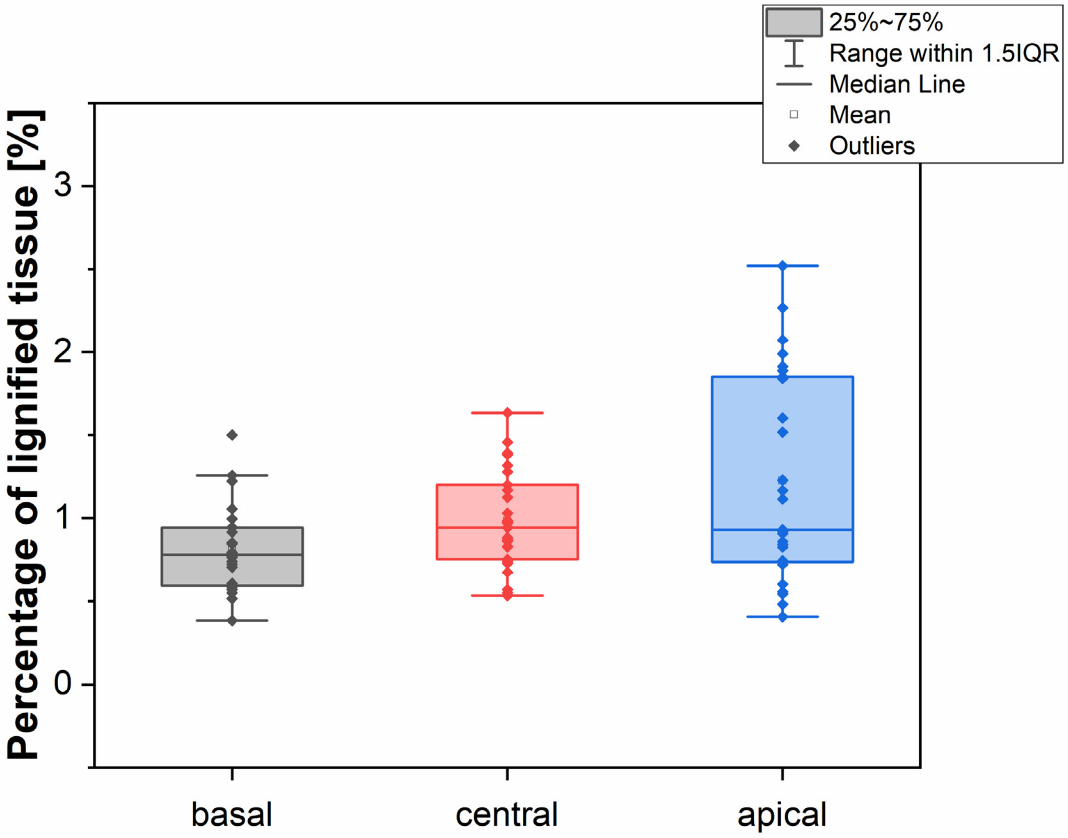

Figure S4: Percentage of lignified petiole tissue of *B. sudjanae* at the basal, central and apical part of the petiole.

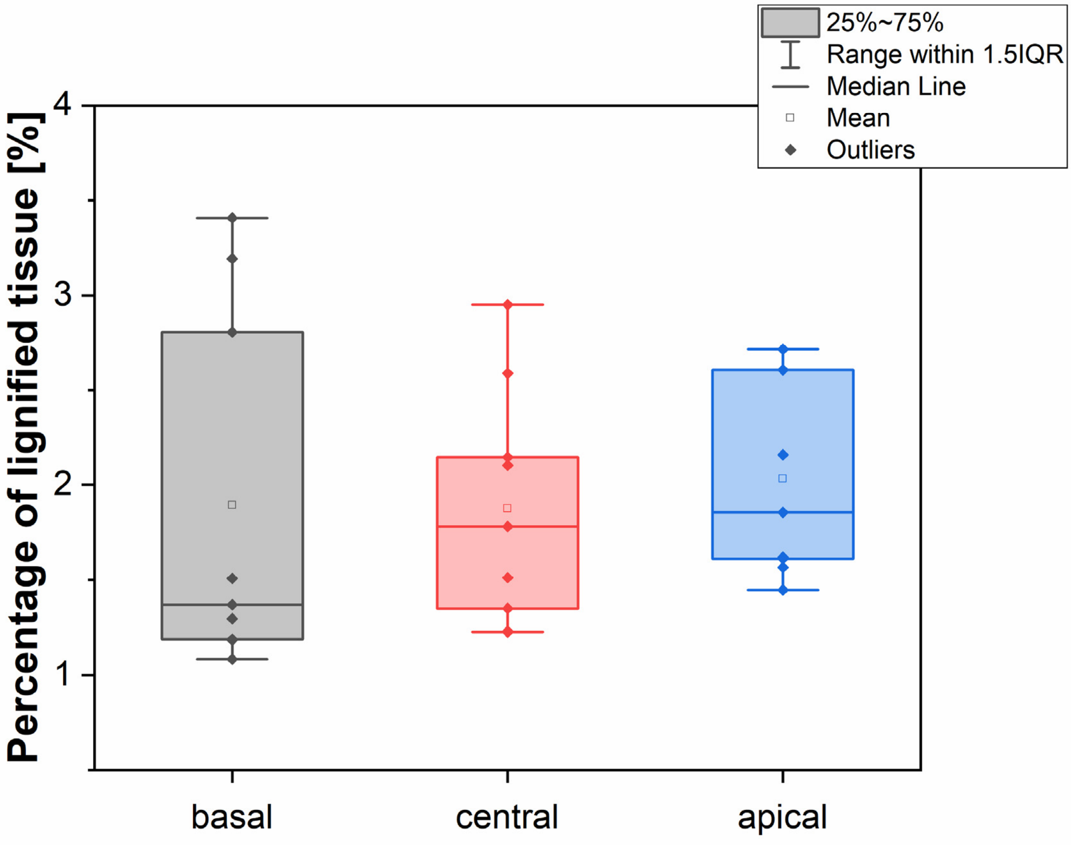

Figure S5: Percentage of lignified petiole tissue of *B. amphioxus* at the basal, central, and apical part of the petiole.

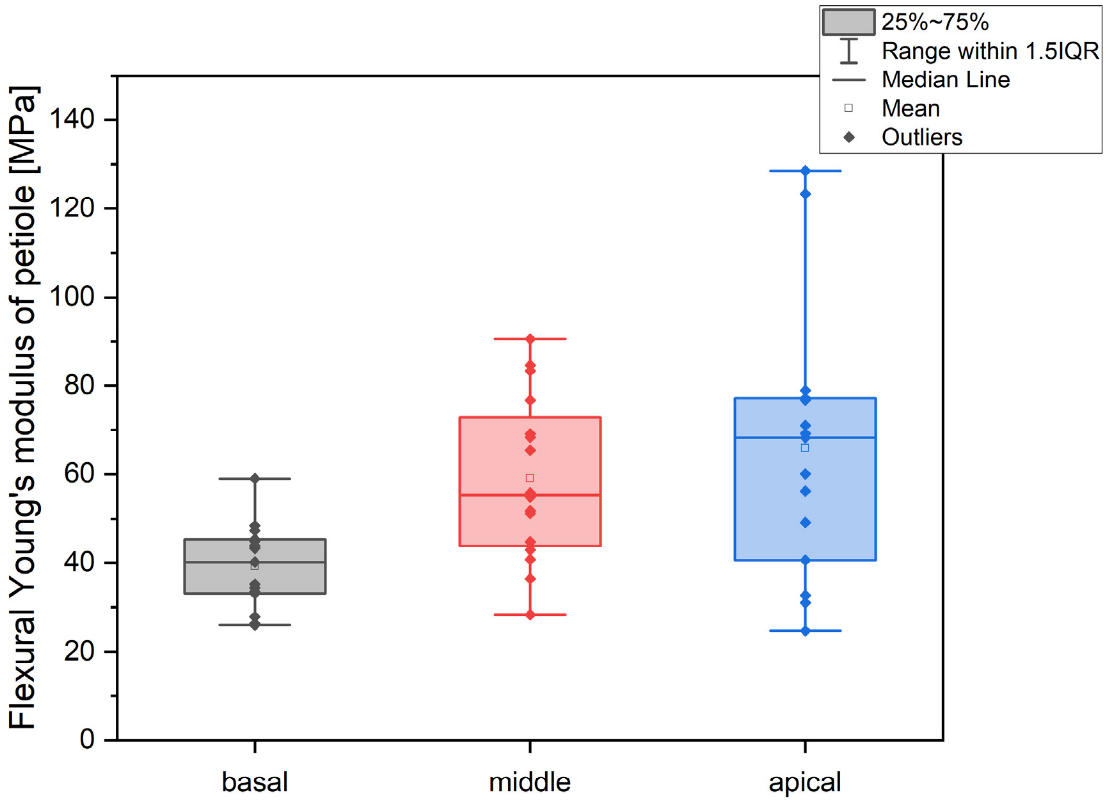

Figure S6: Flexural Young's modulus in MPa of *B. sudjanae* at the basal, central and apical part of the petiole.

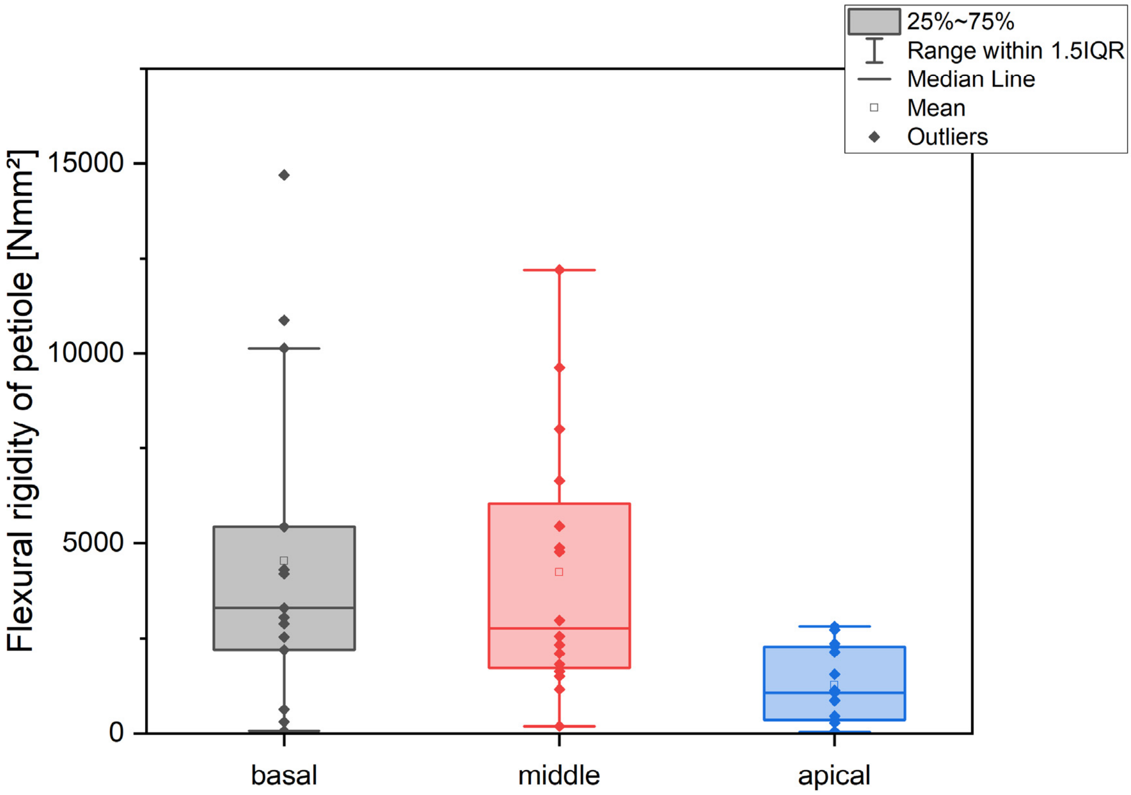

Figure S7: Flexural rigidity in Nmm^2 of *B. sudjanae* at the basal, central and apical part of the petiole.
